# Metatarsophalangeal joint stability: a systematic review on the plantar plate of the lesser toes

**DOI:** 10.1186/s13047-016-0165-2

**Published:** 2016-08-19

**Authors:** Nico M. G. Maas, Margot van der Grinten, Wichor M. Bramer, Gert-Jan Kleinrensink

**Affiliations:** 1 Department of Neuroscience, Erasmus University Medical Centre, P.O. Box 2040, Ee-177, 3000 CA Rotterdam, The Netherlands; 2 Department of Orthopaedic Surgery, Erasmus University Medical Centre, Rotterdam, The Netherlands; 3 Medical Library, Erasmus University Medical Centre, Rotterdam, The Netherlands

**Keywords:** Plantar plate, Metatarsophalangeal stability, Lesser metatarsophalangeal joints, Systematic review

## Abstract

**Background:**

Instability of the metatarsophalangeal (MTP) joints of the lesser toes (digiti 2–5) is increasingly being treated by repair of the plantar plate (PP). This systematic review examines the anatomy of the plantar plate of the lesser toes, and the relation between the integrity of the plantar plates of the lesser toes and lesser MTP joint stability.

**Methods:**

The databases of Embase.com, Medline (Ovid), Web of Science, Scopus, Cochrane, Pubmed not medline, Cinahl (ebsco), ProQuest, Lilacs, Scielo and Google Scholar were searched in June 2015 from inception. Studies were included if they were in English, contained primary data, and had a focus on plantar plate anatomy of the lesser toes or on the relationship between integrity of the plantar plate and MTP joint (in)stability. Study characteristics were extracted into two main tables and descriptive anatomical and histological data were summarized into one schematic 3D drawing of the plantar plate.

**Results:**

Nine studies were included in this systematic review, of which five addressed plantar plate anatomy as such and four focused directly and indirectly on plantar plate integrity related to MTP joint stability.

**Conclusion:**

This is the first systematic review regarding plantar plate anatomy related to MTP joint stability of the lesser toes. This review iterates the importance of plantar plate anatomy and integrity for MTP joint stability, and it delineates the lack of primary data regarding plantar plate anatomy of the lesser toes and MTP joint stability.

**Electronic supplementary material:**

The online version of this article (doi:10.1186/s13047-016-0165-2) contains supplementary material, which is available to authorized users.

## Background

Further specialization in orthopaedic and podiatric surgery has, amongst others, led to an increased attention for anatomical details, including the interest in the pathophysiology and mechanism of pain beneath the metatarsal heads (metatarsalgia) [1–3]. Metatarsalgia symptoms such as gradual onset of forefoot pain, edema and a positive drawer sign can be explained by instability of the metatarsophalangeal (MTP) joint [4, 5], MTP joint instability is described as a dorsal subluxation or dislocation of the base of the proximal phalanx over the metatarsal head. The traditional etiology of instability of the lesser MTP joints in the sagittal and/or transverse plane is described in the literature by plantar plate degeneration and rupture [6, 7]. Alternative causes described in literature include attenuation of the collateral ligaments and the deep transverse metatarsal ligament, and capsular degeneration [8, 9]. The first choice in treating instability of the MTP joints is conservative treatment and is accomplished with shoe wear modifications, metatarsal pads, and custom-made orthoses [5]. Operative treatment may consist of an indirect reconstruction of the MTP joint in which the toe is realigned without reconstruction of the plantar plate [6, 10]. Lately, studies were published in which a direct repair of the plantar plate is reported [6, 11–13]. To be able to determine the best treatment of metatarsalgia it is paramount to know the anatomy (normal and pathologic) of the MTP joints. In recent publications the anatomy of the plantar plate is described. However, only a few authors addressed the relationship between plantar plate integrity and stability. Furthermore, no systematic review on the anatomy and mechanics of the plantar plate has been published. The biomechanics of the first MTP joint are different from the lesser toes. This difference is caused by anatomical differences e.g. the sesamoids, the abductor and adductor muscles, the position in the foot and a larger metatarsal head. Therefore, only articles concerning the lesser MTP joints were included.

The present systematic review has two objectives. Firstly, it assesses and elucidates the published literature regarding the anatomy of the plantar plate of the lesser toes. Secondly, it reviews the literature about the relationship between the integrity of the lesser plantar plates and MTP joint stability.

## Methods

### Literature search

The databases of Embase.com, Medline (Ovid), Web of Science, Scopus, Cochrane, Pubmed (recent articles, not yet indexed by medline), Cinahl (Ebscohost), ProQuest, Lilacs, Scielo and Google Scholar were searched from inception until June 2015. The literature search was designed by an experienced biomedical information specialist (WB). The search combined synonyms for: plantar plate with synonyms for injury, or rupture, and terms for joint, or metatarsalgia. When available, thesaurus terms were used (such as Mesh in medline, and Emtree in Embase) and combined with words in title and/or abstract. An overview of the complete electronic search for all databases is shown in Additional file 1.

### Study selection

Two review authors (NM and MG) independently screened the citations from the searches, and decided which full text articles should be retrieved. They then independently applied inclusion and exclusion criteria resolving any differences in opinion through discussion. Articles met the inclusion criteria if they were in English, contained primary data, and had a focus on the anatomy of the plantar plate of the lesser toes and/or metatarsophalangeal joint (in)stability of the lesser toes.

**Fig. 1:**
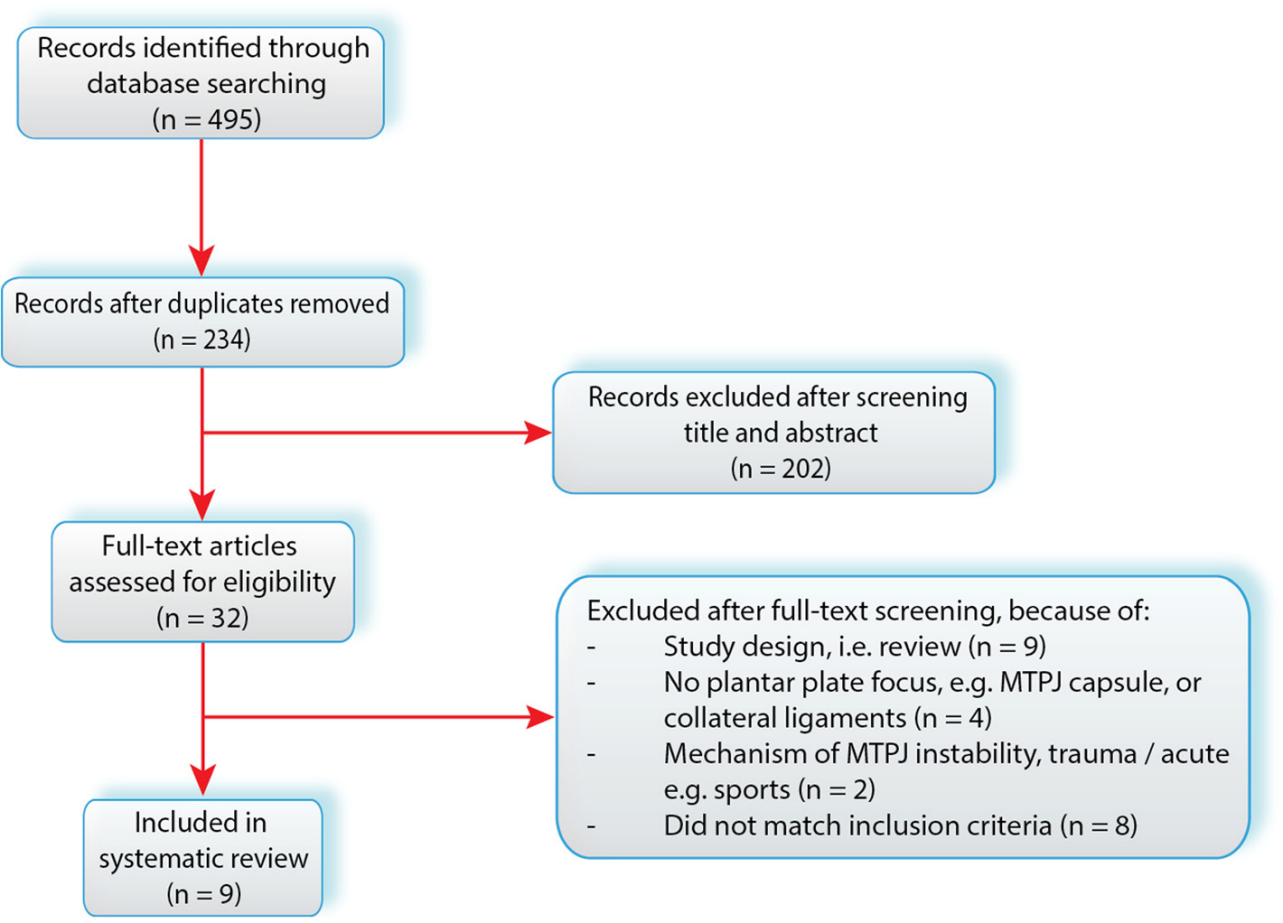
PRISMA flow diagram [21]

Additional citation tracking was performed by manual screening of the reference lists of the eligible studies. A third author (GJK) was available to ultimately decide whether a study should be included or excluded if agreement could not be reached. For exclusion criteria, see Results section (Fig. 1).

### Data extraction & synthesis

**Fig. 2:**
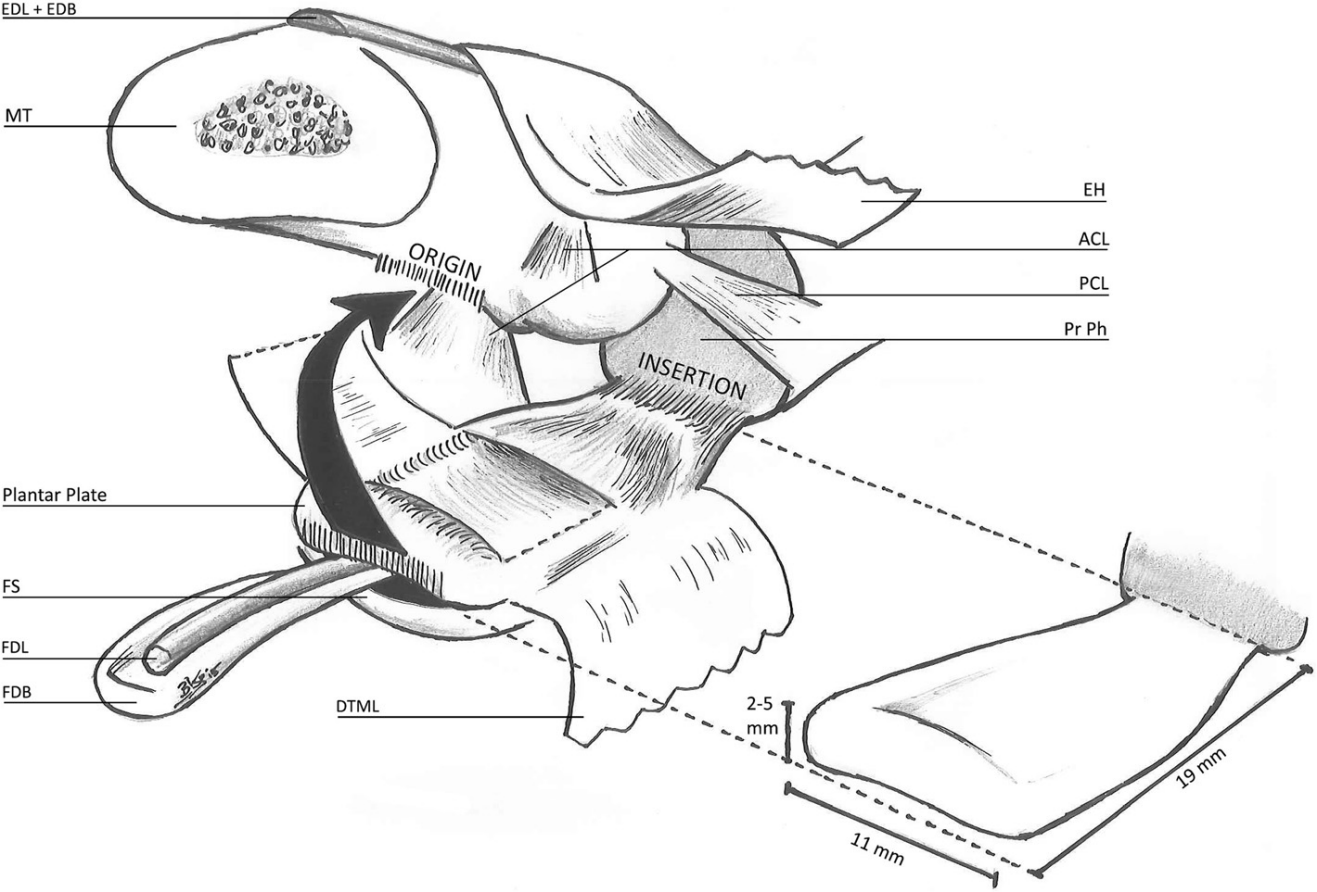
Descriptive anatomical & histological data visualized in a schematic 3D drawing of the PP. Legend: *EDL+ EDB*, extensor digitorum longus/brevis; *MT*, metatarsal; *EH*, extensor hood; *ACL*, accessory collateral ligament; *PCL*, proper collateral ligament; *Pr Ph*, proximal phalanx; *FS*, flexor sheath; *FDL + FDB*, flexor digitorum longus/brevis; *DTML*, deep transverse metatarsal ligament

Outcome data for each included study were extracted by NM. As a result of a lack of pre-tested data forms or quality criteria for reviewing studies performed on anatomic specimens, we did not perform a quality assessment for the anatomical studies. Data are summarized in Table 1 (detailed version in Appendix 1) and Table 2 (detailed version in Appendix 2), and visualized into a schematic drawing (Fig. 2).

**Table 1:** Overview of articles (anatomical studies) focusing on plantar plate anatomy

Author; year	Specimen: lesser MTPJs	Age; Gender	PP dimensions
Johnston, Smith [15]	20 FF of 5 cadavers	NR	L: mean 19 mm; 2^nd^ MTPJ 16–23 mm W: mean 11 mm proximally, to 9 mm distally; 2^nd^ MTPJ 8–13 mm T: 2–5 mm
Deland, Lee [8]	30 FF of 6 cadavers	Median age 64 y; gender NR	L: mean 18.8 mm 2^nd^, 3^rd^ MTPJ: 20 mm mean 4^th^, 5^th^ MTPJ: 17 mm mean W: NR T: 2–4 mm
Deland and Sung [17]	8 FF of 1 cadaver (†, ‡)	68 y, F	NR
Gregg, Marks [14]	8 FF of 1 cadaver	19 y, F	Referring to Johnston et al.
	8 soft-embalmed of 3 cadavers	Age NR; 2x M, 1x F	
Coughlin, Schutt [22]	16 FF cadaveric specimens, ‡	Age NR; 10x F 2x M 4x NR	NR

Legend: *FF* fresh-frozen, *L* length, *W* width, *T* thickness, *y* years, *NR* not reported, *F* female, *M* male

†With hallux valgus

‡With 2nd crossover toe

**Table 2:** Overview of articles (anatomical studies) regarding PP integrity and MTP joint stability

Author; year	Purpose	Specimen	Results	Conclusion
Bhatia [16]	Measure *dorsal* displacement of the proximal phalanx, to determine the anatomical restraints to dislocate the 2^nd^ MTPJ.	25 FF cadaveric 2^nd^ MTPJs	Mean force required to dislocate the toe, after division of, PP: 26 N; both collateral ligaments: 20 N. Division of PP + collateral ligaments created an unstable joint dislocating at 8 N.	Plantar plate and the collateral ligaments are the main stabilizers of the MTPJ.
Cooper and Coughlin [6]	Measure total distraction of metatarsal head and proximal phalanx base, to elucidate the necessary dissection for best plantar plate exposure.	8 FF cadaveric 2^nd^ MTPJs	Dorsal capsulotomy of the 2^nd^ MTP joint with collateral ligament release off of the proximal phalanx base, combined with a subcapital oblique metatarsal osteotomy provided an average 8–8.5 mm exposure.	Plantar plate exposure is best obtained by releasing collateral ligaments off of proximal phalanx with a subcapital oblique osteotomy.
Suero, Meyers [5]	Measure and compare *dorsal* displacement of the proximal phalanx, whilst isolated and combined sectioning of PP and surrounding structures.	54 FF cadaveric 2^nd^, 3^rd^ and 4^th^ MTPJs	Mean dorsal displacement of intact MTPJ: 10.6 mm. Dorsal displacement increased in % after sectioning: PP: 19 % MCL + LCL: 37 % PP + MCL + LCL: 63 %	Plantar plate is main isolated restraint for dorsal MTP joint translation.
Chalayon [18]	Compare intact sagittal plane stability (superior subluxation, dorsiflexion, plantarflexion) of lesser MTP joint of the 2^nd^, 3^rd^ and 4^th^ toes, and to quantify the role of PP for controlling sagittal plane stability.	4 FF cadaveric 2^nd^, 3^rd^ and 4^th^ MTPJs	No significant differences measurable between the stability of intact lesser toes. Overall mean stability of lesser MTPJs: superior subluxation: 3.03 ± 0.93 N/mm, dorsiflexion: 2.07 ± 0.38 N/mm; plantarflexion: 0.42 ± 0.06 N/mm. Disruption of PP significantly (*P* ≤ 0.001; pooled sample size of twelve) decreased stability (in %) to overall mean stability by an average in: subluxation: 23 ± 5 %, dorsiflexion: 34 ± 9 %, plantarflexion: 26 ± 11 %	PP contributes significantly to sagittal plane stability of the lesser MTP joints.

Legend: *FF* fresh-frozen, *EH* extensor hood, *MCL* medial collateral ligament, *LCL* lateral collateral ligament

## Results

### Search results

The initial search yielded 495 records. After removing duplicates, 234 articles were screened based on title and abstract. Full text articles of thirty-two studies were retrieved. Nine studies were included, of which five addressed plantar plate anatomy as such and four focused directly and indirectly on plantar plate integrity related to MTP joint stability. Additional citation tracking resulted in no relevant articles to be included and for study selection, agreement was reached by NM & MG, therefore a decisive role for GJK was unnecessary. The study selection is visualized with a flow diagram (Fig. 1) also containing the exclusion criteria.

#### Plantar plate anatomy

##### Morphology

The plantar plate is a broad ribbon-like disc, firm but flexible, with a form ranging from rectangular to trapezoidal [8, 14]. Its plantar surface is smooth, and grooved at its outer borders to provide a gliding plane for the flexor tendons [8, 15]. Its vessels appear to enter peripherally, mainly plantarly, and are part of the loose connective tissue septae (endotenon) that surround the collagen fascicles [14].

The periosteum of the metatarsal shaft is the origin of the plantar plate. Although Johnston et al. [15] states that the portion of plantar plate that lies just below metatarsal head is both thicker and broader than the distal attachment; others, report the origin of the plantar plate to be thin, fibrous and of synovial type [8]. Moreover, the strength and thickness of the origin was found greater at the periphery [15]. The plantar plate has its insertion firmly and directly into bone on the plantar surface of the proximal phalanx. This seems to be the strongest attachment, with the full thickness of the plate inserting into the bone [6, 8]. The enthesis reveals longitudinal and interwoven collagen bundles entering the proximal phalanx with multiple interdigitations [14]. The distinct thickening of the tendon sheath is adherent or invested into the borders of the plantar plate and represents a flexor tendon pulley [14].

The thickest portion of the plate was its midportion distal to its metatarsal origin and proximal to the phalangeal insertion. At the phalangeal base, the borders are thicker than centrally; proximal to the phalangeal base there is little difference between the thickness at the borders and the central portion [14].

##### Histology & composition

Bright-field microscopic evaluation showed the plantar aspect of the plantar plate to have a rather distinct cartilage matrix. However it becomes more ligament-like the further it distally tapers, containing fewer chondrocytes and a greater abundance of fibroblasts. Therefore, it is mostly known as fibrocartilage [14]. From immuno-histochemical examination, the collagen was identified as primarily type I, the same type and composition found in other fibrocartilage structures like the meniscus in the knee and the annulus fibrosis of the intervertebral disc of the spine [8, 15]. No elastin fibers were found by Deland et. [8], however small concentrations of elastin (stains black in elastin stain) were found by Johnston et al. [15].

Collagen bundles both in the central and peripheral part, were in the region of 20 μm thick [14]. In polarized light, they are oriented longitudinally in the dorsal two thirds of the plate, as visualized in Fig. 2 [8]. In the plantar one third, the fibers were oriented transversely at the level of the deep transverse metatarsal ligament (DTML) and were continuous with it, merging with the collateral ligaments [14].

##### Plantar plate surroundings

The plantar plate functions as the insertion point of tendons, ligaments, and stabilizing structures. The DTML is attached to the medial and lateral borders of the plantar plate. The flexor digitorum longus and brevis tendons lie in their sheath plantar to the plantar plate. The four lumbrical muscles arise from the medial border of the flexor digitorum longus tendons and insert to the medial side of the proximal phalanx on the plantar surface and insert into the distal plantar aspect of the plantar plate. The tendons of the plantar interossei muscles lie just dorsal to the DTML and run a course adjacent to the plantar plate, with some fibers of the interossei inserting directly into the plantar plate. The flexor digiti minimi brevis and abductor digiti minimi tendons both insert on the plantar aspect of the fifth toe on the fibular side just distal to the plantar plate. Some fibers of these tendons insert into the plantar plate [15].

Interestingly, Deland et al. [8] found a ligamentous band distinct from the plate or collateral ligaments at the plate’s insertion. This band originated from the distal lateral portion of the plate and inserted onto a tubercle at the lateral-plantar portion of the proximal phalanx. The location of its insertion on the proximal phalanx was superficial to the attachments of the collateral ligament and interosseous tendons. Its dimensions (mean length 3 to 4 mm and thickness less than 0.5 mm) showed it to be considerably smaller and less substantial than the plate or collateral ligaments.

#### Plantar plate integrity and MTP joint stability

With an intact plantar plate and normal MTP joint, the toes normally dorsiflex passively and the fat pad of the metatarsal head moves with the plantar plate to cover the head of the metatarsal as a shock-absorbing cushion. In the foot, *dynamic* stabilization is provided by the extrinsic and intrinsic musculature, where *static* stabilization is mainly provided by the plantar plate [14, 16]. When compared to normal joints, pathologic MTP joints will allow excessive dorsal translation of the proximal phalanx [5]. Chronic injury to the plantar plate causes attritional and adaptive changes in the plantar plate (elongation, attenuation and eventually rupturing), capsule, ligaments, and intrinsic tendons, which results in dislocation and instability of the MTP joints [6, 16, 17]. With rupture of the plantar plate, the proximal phalanx assumes a dorsally subluxed position [14]. Hereby the extensor tendons cannot extend the proximal and distal interphalangeal joints and over time the plantar plate and the flexor tendons tend to shift dorso-medially [17].

To date, in four studies the direct and indirect relation of plantar plate integrity and MTP joint stability was described (see Table 2). Bhatia et al. [16] and Suero et al. [5] used vertical forces measuring vertical translation. Cooper et al. [6] used horizontal measurement of total distraction of the metatarsal head and proximal phalanx base. Chalayon et al. [18] created a different test model. Instead of disarticulating the feet at the ankle and amputating the first and third toes, they dissected above the origin of the flexor digitorum longus tendon, permitting natural rotatory dorsiflexion testing or simulation of the clinical dorsal drawer test.

As Bhatia et al. and Chalayon mentioned, when vertical forces are applied in a static stabilization, this may not represent the dynamic forces produced during the normal motion of the MTP joint [16, 18]. Also Suero et al. [5] elucidates the role of each individual static stabilizing structure by sequentially sectioning structures and putting it to the vertical test. As a result, Bhatia et al., Suero et al. and Chalayon et al. conclude, the plantar plate and the collateral ligaments are the main stabilizers, with the plantar plate being the main restraint for dorsal MTP joint translation and significantly contributes to sagittal stability [5, 16, 18].

## Conclusion

Recently the role of repair of lesions of the plantar plate in relation to MTP instability and metatarsalgia is subject of increased interest. When trying to find solutions for clinical problems, knowing the anatomy and its function is of uttermost importance. The subject of this systematic review is the anatomy of the plantar plate of the lesser toes and the relation between the integrity of the plantar plates of the lesser toes and MTP joint stability.

The plantar plate is a firm and flexible disc with a form, varying from rectangular to trapezoidal. The thickness ranges from 2 to 5 mm, the length from 16 to 23 mm, and the width 8 to 13 mm. Its plantar surface is smooth, and is grooved at its outer borders to provide a gliding plane for the flexor tendons. From the (histo-)anatomic information, and in according to Pauwel’s theory of ‘causal histogenesis’ that collagen fibrils are always oriented in the direction of the greatest tension, one can draw three conclusions [14].

Firstly, the plantar insertional fibers withstand mostly *tensile* forces, by providing support to the windlass mechanism with the insertion of the plantar fascia [8, 14]. In addition, the dorsal-to-mid insertional fibers experience also tensile forces [14]. Secondly, it withstands *compressive* loads acting as a cushion through its fibrocartilage structure.

Thirdly, it assists in MTP joint *stability* through its central location and attachments to many surrounding structures. Moreover, plantar plate injury or combined injuries to the plate, with the extensor hood and the collateral ligaments have shown to cause significant instability, just as the plantar plate has proven to be the most important isolated sagittal stabilizer of the MTP joint [5, 18]. With plantar plate injury, support is lost, contributing or leading to metatarsalgia.

In the context of current evidence, this is the first systematic review regarding plantar plate anatomy related to MTP joint stability of the lesser toes. To date the direct relation between plantar plate lesions and MTP joint instability is still controversial. It stands to reason that once a soft tissue structure, i.e. the collateral ligament or plantar plate undergoes attritional and adaptive changes due to chronic injury, it will in turn lead to deformation and attenuation, resulting in MTP joint instability and/or metatarsalgia [8, 14, 19]. The adaptive changes can be due to various causes, e.g. chronic hyperextension or chronic synovitis, eventually leading to loss of soft tissue balance [20].

In our opinion, at least, it is challenging to prove MTP joint instability to be caused directly by plantar plate injury, as MTP joint instability is not pathognomonic for plantar plate rupture.

This review showed that only Suero et al., Bhatia et al. and Chalayon et al. have demonstrated, in an in vitro anatomical study, that plantar plate injury in itself can cause MTP joint instability, as isolated or combined sectioning of the plantar plate showed significant instability [5, 16, 18]. Caution is advised when using this knowledge in cases with *chronic* instability, as a model was created simulating *acute* instability of the lesser toes, which cannot account for biological healing as may be seen in chronic situations [18]. Although much is published regarding plantar plate repair techniques, scarce primary data regarding details of normal plantar plate anatomy of the lesser toes is available in cases of metatarsalgia or instability. Remarkably, only two studies have described the lesser plantar plate dimensions, of which one reference of Deland et al. (1995) was referred to as ‘being checked’ [8, 15]. Furthermore, only a few studies have addressed the relation of plantar plate injury and normal MTP joint mechanics and stability of the lesser toes [5, 6, 16, 18].

We attempted to conduct a quality assessment of the included studies, however, it was impracticable due to lack of pre-tested data forms or quality criteria for reviewing studies performed on anatomical specimen. Therefore, we could not perform a standardized critical appraisal. To improve future transparency and quality assessment, we recommend a guideline or quality criteria for reporting (biomechanical) anatomical studies.

We report no geographical or temporal constraints, and strived to minimize our source and publication bias. Only articles in English and published in databased literature were included, creating a scope bias. In addition to the existing literature, we recommend a study re-examining the anatomical dimensions in normal and pathological MTP-joints of the lesser toes, the incidence of plantar plate injury and the relationship between instability, plantar plate injury and metatarsalgia. Furthermore, we are interested in the relationship between a functional instable first ray and plantar plate injury. This can be a topic of further research.

This study shows the lack of primary data regarding plantar plate anatomy of the lesser toes and MTP joint stability. Nevertheless, we endeavoured to elucidate and clarify the importance of plantar plate anatomy and integrity, to provide the necessary building blocks for clinical practice and future research.

## Appendix 1

**Table 3:** Detailed overview of articles regarding plantar plate anatomy

Author	Purpose	Specimen: lesser MTP joints	Details: age, sex, pathology	Measurement	Method	PP dimensions
Johnston, et al. (1994) ^10^	Describing macro- and microscopic anatomy of the PP of the MTP- and PIP joints, and correlate with spontaneous lesser toe instability.	5 FF	NR Medical history checked^a^	Metric ruler for dimensions Microscopic examination Biochemical analysis	Dissection (using 2.5x loupes) Paraffin embedded (on a microtome to 4 μm), stained with trichrome, hematoxylin and eosin, and elastin.	**L**: 2^nd^ MTP joint 16–23 mm (mean 19 mm) **W**: 2^nd^ MTP joint 8–13 mm (mean 11 mm proximally to 9 mm distally) **T**: 2–5 mm
Deland, et al. (1995) ^6^	Detailed description about the PP, including dimensions, attachments and histology.	30 FF lesser MTP joints of 6 cadavers	Median age 46 y; range: 25–79 y Sex: NR X-ray + macroscopic inspection	Micrometer for dimensions Microscopic examination Immunohisto- chemical analysis	Dissection, removing entire ray en bloc. Separate feet were sliced in transverse and sagittal planes. Formalin fixed, paraffin embedded (cut at 5 μm thickness), stained with eosin and hematoxylin.	**L**: 18.8 mm mean (2^nd^, 3^rd^ MTP joint: 20 mm mean) (4^th^, 5^th^ MTP joint: 17 mm mean) **W**: NR **T**: 2–4 mm
Deland and Sung (2000) ^7^	Dissecting a full crossover toe for a better understanding of the pathologic anatomy of a medial crossover toe.	8 FF lesser MTP joints of 1 cadaver (with hallux valgus and second medial crossover toe)	68 y, female X-ray + macroscopic inspection Medical history checked^b^	Micrometer for dimensions	Layer by layer dissection and comparing with opposite foot (with normally aligned 2^nd^ MTP joint).	NR
Gregg, et al. (2007) ^9^	Develop a more complete understanding of the morphology of the PP and the junction between fibrocartilage and bone.	8 FF lesser MTP joints of 1 cadaver & 8 soft-embalmed lesser MTP joints of 3 cadavers (2^nd^ MTP joints: 5; 3^rd^ MTP joint: 1; 4^th^ MTP joints: 2)	19 y, female & 2 male, 1 female (range 74–92 y)	Magnification: x1.2; x20 Bright-field and Polarized light microscopy	Dorsal soft tissues were removed adjacent to the MTP Joints and the extensor tendons severed. PP attachments were carefully severed. 10 % Formalin fixed, decalcified with EDTA and formic acid, paraffin (10 μm) embedded, stained with Masson’s trichrome, haematoxylin, eosin and alcian blue.	NR (Referring to Johnston)
Coughlin, et al. (2012) ^5^	Document the presence and pattern of PP tears	16 fresh-frozen (below-knee) cadaveric specimens with clinical diagnosis 2^nd^ crossover toe	4 specimen gender unknown; 10/12 was female (range 70–95 y)	Demographics Simulated weight bearing Radiographs: obtained prior to dissection	Extirpation of the 2^nd^ MT head, careful inspection of the PP and collateral ligaments of each joint.	NR

Legend: *FF* fresh-frozen, *NR* not reported, *L* length, *W* width, *T* thickness, *PP* plantar plate, *PIP* Proximal Interphalangeal, *MTP* metatarsophalangeal, *y* years

^a^For no pre-existing diabetes, peripheral vascular disease or rheumatologic disorders

^b^No history of rheumatoid arthritis or other inflammatory disease, died of myocardial infarction

## Appendix 2

**Table 4:** Detailed overview of articles regarding plantar plate integrity and MTP joint stability

Author	Purpose	Specimen	(Fixating) Technique	Outcome measure	Results	Conclusion
Bhatia (1994) ^1^	Determine anatomical restraints to dislocation of the 2nd MTP joint and assess biomechanical efficacy for a common stabilizing technique.	25 FF cadaveric feet (disarticulated at ankle; long flexor tendons preserved at medial malleolus)	Universal testing machine: Bionix 858, MTS systems Minneapolis, Minnesota. Hindfoot was transfixed with an aluminum jig. A nylon block was secured to dorsum of the 2^nd^ metatarsal and a K-wire passed transversely through the proximal phalanx. Each load cycle was repeated 4x.	Vertical load, measuring dorsal displacement of the proximal phalanx. 5 N preload applied, followed by a constant vertical displacement of 2 mm/min. Load–displacement curves were measured.	Mean force required to dislocate the toe was: Group A: 26 N (SD 5.32; 22–34 N) after division of the PP Group B: 20 N (SD 3.5 N; 15–23 N) after division of the collateral ligaments Group C: Division of both the PP and collateral ligaments created an unstable joint that dislocated at an applied mean load of 8 N (SD ± 4.74; 5–10 N)	The collateral ligaments and the PP are the main stabilizers of the MTP joint.
Cooper and Coughlin (2011) ^4^	Elucidate the necessary dissection toe expose and potentially repair lesions of the PP trough a dorsal approach.	8 FF cadaveric specimen (below the knee amputated, examined range of motion & deformity)	4 cm incision dorsal approach; extensor tendon z-tenotomy with dorsal capsulotomy. Released from base of proximal phalanx vs released from metatarsal head McGlamry metatarsal head release and Weil osteotomy vs Weil osteotomy	(Horizontal) Measurement of total distraction of metatarsal head and proximal phalanx base. After each step fluoroscopic and digital photography to determine amount of exposure of plantar plate gained by each step.	Dorsal capsulotomy of the 2nd MTP joint with collateral ligament release off of the proximal phalanx base, then combined with a subcapital oblique metatarsal osteotomy provided an average 8–8.5 mm exposure. Little improvement of visualization of the plantar plate after use of theMcGlamry elevator for plantar release.	PP exposure is best obtained by releasing collateral ligaments off of proximal phalanx in combination with a subcapital oblique osteotomy.
Suero, et al. (2012) ^20^	Hypothesis: isolated sectioning of the PP would result in greater dorsal translation compared to isolated sectioning of the MCL, LCL, or EH, and that combined injury to two or more structures would result in greater dorsal translation compared to isolated PP injury.	54 FF toes of 18 cadavers (from which the 2^nd^, 3^rd^ and 4^th^ toes were resected at base of metatarsal)	Two most distal phalanges were excised. Potted phalangeal end of the specimen was affixed to a clamp, attached to the load cell of a hydraulic load frame (MTS Systems Copr., Eden Prairie, MN) Potted base of metatarsal attached to the piston of the load frame.	Vertical load, measuring dorsal translation of the proximal phalanx. 30 N axial load in plantar-dorsal direction, 0.5 N/sec	Mean dorsal displacement for intact MTP joint: 10.6 mm (SD 3.03 mm) Mean dorsal displacement after sectioning: PP: 19 % (SD 13 %)^a^ EH:11 % (SD 5 %) MCL: 13 % (SD 7 %) LCL: 17 % (SD 7 %) MCL + LCL: 37 % (SD 18 %)^a^ EH + MCL + LCL: 45 % PP + MCL + LCL: 63 % (SD 54 %)^a^	PP is main restraint for dorsal MTP joint translation. Injury to the plantar plate or combined injuries to the plate, EH, MCL, LCL appear to cause significant instability, suggesting a more aggressive treatment.
Chalayon, et al. (2013) ^3^	Compare intact sagittal plane stability (superior subluxation, dorsiflexion, plantarflexion) of lesser MTP joints. Quantify role of PP for sagittal plane stability.	4 FF cadaveric specimen (3 male, death range 48–63 y) (transected ± 25 cm proximal to the plantar surface of the foot	-Delrin mounting plate, allowing unencumbered plantarflexion motion, secured to calcaneus and secured to 2.54 cm thick aluminum platform mounted to base plate of materials testing system. Two custom fixers designed and fabricated from ABS plastic using a rapid prototype machine; and aluminum wire, 18-gauge, drilled in bone and into fixer.	Each specimen was displaced to 80 % of determined physiological range, and load data were recorded at 100 % of full physiological motion for each motion axis. Displacing with a 25-mm moment arm measured distally from the center of rotation of lesser MTPJ.	No significant differences measurable between the stability of intact lesser toes. Overall mean stability of intact lesser MTPJs:Superior subluxation: 3.03 ± 0.93 N/mm Dorsiflexion: 2.07 ± 0.38 N/mm Plantarflexion: 0.42 ± 0.06 N/mm. Disruption of PP significantly (*P* ≤ 0.001) decreased stability by an average in: Subluxation: 23 ± 5 % Dorsiflexion: 34 ± 9 % Plantarflexion: 26 ± 11 %	Data from this study indicated that PP significantly contributed to the sagittal plane stability of the lesser MTP joints.

Legend: *FF* fresh-frozen, *PP* plantar plate, *EH* Extensor Hood, *MCL* medial collateral ligament, *LCL* lateral collateral ligament, *MTP* metatarsophalangeal

^a^Significant finding

## Additional file

Additional file 1: Search strategy 2 June 2015. (DOCX 15 kb)

## Abbreviations

†: With hallux valgus
‡: With 2nd crossover toe
ACL: Accessory collateral ligament
DTML: Deep transverse metatarsal ligament
EDL/EDB: Extensor digitorum longus/brevis
EH: Extensor hood
F: Female
FDP/FDS: Flexor digitorum longus/brevis
FF: Fresh-frozen
FS: Flexor sheath
L: Length
M: Male
MT: Metatarsal
MTP: Metatarsophalangeal
MTPJ: Metatarsophalangeal joint
NR: Not reported
PCL: Proper collateral ligament
PP: plantar plate
Pr Ph: Proximal phalanx
T: Thickness
W: Width
y: Years
