# Supplementary material for: Metatarsophalangeal joint stability: a systematic review on the plantar plate of the lesser toes
Source: J Foot Ankle Res. 2016 Aug 19;9:32. doi: 10.1186/s13047-016-0165-2 (PMC4992309; doi:10.1186/s13047-016-0165-2)
Supplement: Additional file 1: — Search strategy 2 June 2015. (DOCX 15 kb) [file 13047_2016_165_MOESM1_ESM.docx]

Additional file 1: Search strategy 2 June 2015

|  | Before deduplication | After deduplication |
| --- | --- | --- |
| embase.com | 101 | 101 |
| Medline (OvidSP) | 70 | 4 |
| Web-of-science | 79 | 26 |
| Scopus | 117 | 26 |
| Cinahl (ebsco) | 25 | 1 |
| PubMed not medline | 16 | 15 |
| Cochrane | 0 | 0 |
| ProQuest | 2 | 2 |
| Lilacs | 1 | 1 |
| Scielo | 1 | 1 |
| Google Scholar | 100 | 69 |
| **Total** | **495** | **234** |

**embase.com**

((plantar* NEAR/3 (plate* OR ligament* OR capsul*)):ab,ti AND (injury/exp OR (injur* OR rupture* OR tear* OR lesion* OR sprain* OR strain* OR dislocat* OR predislocat* OR attenuat* OR insufficien* OR disruption* OR avuls* OR repair* OR advance*):ab,ti) OR (turf NEXT/1 toe*):ab,ti) AND ('joint instability'/de OR metatarsalgia/exp OR (instab* OR stabil* OR metatarsalg*):ab,ti)

**Medline (Ovid)**

((plantar* ADJ3 (plate* OR ligament* OR capsul*)).ab,ti. AND (exp "Wounds and Injuries"/ OR (injur* OR rupture* OR tear* OR lesion* OR sprain* OR strain* OR dislocat* OR predislocat* OR attenuat* OR insufficien* OR disruption* OR avuls*).ab,ti.) OR (turf adj toe*).ab,ti.) AND (exp "joint instability"/ OR metatarsalgia/ OR (instab* OR stabil* OR metatarsalg*).ab,ti.)

**Cinahl (ebsco)**

((plantar* N3 (plate* OR ligament* OR capsul*)) AND (MH "Wounds and Injuries+" OR (injur* OR rupture* OR tear* OR lesion* OR sprain* OR strain* OR dislocat* OR predislocat* OR attenuat* OR insufficien* OR disruption* OR avuls*)) OR (turf N1 toe*):ab,ti) AND (MH "joint instability+" OR MH metatarsalgia+ OR (instab* OR stabil* OR metatarsalg*))

**Cochrane**

(plantar* NEAR/3 (plate* OR ligament* OR capsul*)):ab,ti AND ((injur* OR rupture* OR tear* OR lesion* OR sprain* OR strain* OR dislocat* OR predislocat* OR attenuat* OR insufficien* OR disruption* OR avuls*):ab,ti) AND ((instab* OR stabil* OR metatarsalg*):ab,ti)

**Web-of-science**

TS=(((plantar* NEAR/3 (plate* OR ligament* OR capsul*)) AND ((injur* OR rupture* OR tear* OR lesion* OR sprain* OR strain* OR dislocat* OR predislocat* OR attenuat* OR insufficien* OR disruption* OR avuls*)) OR (turf NEAR/1 toe*)) AND ((instab* OR stabil* OR metatarsalg*)))

**Scopus**

TITLE-ABS-KEY(((plantar* W/3 (plate* OR ligament* OR capsul*)) AND ((injur* OR rupture* OR tear* OR lesion* OR sprain* OR strain* OR dislocat* OR predislocat* OR attenuat* OR insufficien* OR disruption* OR avuls*)) OR (turf PRE/1 toe*)) AND ((instab* OR stabil* OR metatarsalg*)))

**PubMed not medline**

(plantar*[tiab] AND (plate*[tiab] OR ligament*[tiab] OR capsul*[tiab])) AND ("Wounds and Injuries"[mh] OR (injur*[tiab] OR rupture*[tiab] OR tear*[tiab] OR lesion*[tiab] OR sprain*[tiab] OR strain*[tiab] OR dislocat*[tiab] OR predislocat*[tiab] OR attenuat*[tiab] OR insufficien*[tiab] OR disruption*[tiab] OR avuls*[tiab])) AND ("joint instability"[mh] OR metatarsalgia[mh] OR (instab*[tiab] OR stabil*[tiab] OR metatarsalg*[tiab])) NOT medline[sb]

**Google Scholar**

"plantar plate|ligament|capsula" injury|rupture|ruptures|tear|tears|lesion|lesions|dislocation|insufficiency|disruption|avulsion

**ProQuest**

(ti("plantar plate" OR "plantar ligament" OR "plantar capsula" ) OR ab("plantar plate" OR "plantar ligament" OR "plantar capsula" ) ) AND (ti(injur* OR ruptu* OR tear* OR lesion* OR dislocation* OR insufficien* OR disruption* OR avulsion*) OR ab(injur* OR ruptu* OR tear* OR lesion* OR dislocation* OR insufficien* OR disruption* OR avulsion*))

**Lilacs**

**Scielo**

("plantar plate" OR "plantar ligament" OR "plantar capsula" ) AND (injur* OR ruptu* OR tear* OR lesion* OR dislocation* OR insufficien* OR disruption* OR avulsion*)
